# Supplementary material for: HIVprotI: an integrated web based platform for prediction and design of HIV proteins inhibitors
Source: J Cheminform. 2018 Mar 9;10:12. doi: 10.1186/s13321-018-0266-y (PMC5845081; doi:10.1186/s13321-018-0266-y)
Supplement: Supplementary file 2 — Additional file 2. Source code of HIVProtI web server. [file 13321_2018_266_MOESM2_ESM.zip › HIVprotI_Source-code/hivprotI/weka-3-6-6/doc/weka/estimators/package-frame.html]

weka.estimators


weka.estimators

Interfaces 
  
*ConditionalEstimator*
  
*IncrementalEstimator*

Classes 
  
CheckEstimator
  
CheckEstimator.AttrTypes
  
CheckEstimator.EstTypes
  
DDConditionalEstimator
  
DiscreteEstimator
  
DKConditionalEstimator
  
DNConditionalEstimator
  
Estimator
  
EstimatorUtils
  
KDConditionalEstimator
  
KernelEstimator
  
KKConditionalEstimator
  
MahalanobisEstimator
  
NDConditionalEstimator
  
NNConditionalEstimator
  
NormalEstimator
  
PoissonEstimator
